# Phlegmonous gastritis in a patient with mixed-phenotype acute leukemia in the neutropenia phase during chemotherapy: A case report

**DOI:** 10.1097/MD.0000000000017777

**Published:** 2019-11-11

**Authors:** Dayu Shi, Jinde He, Meng Lv, Rui Liu, Ting Zhao, Qian Jiang

**Affiliations:** a Peking University People's Hospital, a Peking University Institute of Hematology, b Department of Gastroenterology, Beijing; b Department of Gastroenterology, Peking University People's Hospital, Beijing; c Collaborative Innovation Center of Hematology, Soochow University, Suzhou, Jiangsu Province, China.

**Keywords:** antibiotic, mixed-phenotype acute leukemia, phlegmonous gastritis, *Stenotrophomonas maltophilia*

## Abstract

**Rationale:**

Phlegmonous gastritis is a rare bacterial infection of the gastric wall with high mortality. However, diagnosis of phlegmonous gastritis is difficult and standard treatment remains unestablished.

**Patient concerns:**

We report a 33-year-old male patient with mixed-phenotype acute leukemia who developed acute phlegmonous gastritis during the neutropenia phase on induction chemotherapy and was successfully treated.

**Diagnoses:**

The patient was diagnosed with phlegmonous gastritis, which might be caused by *Stenotrophomonas maltophilia* on the basis of clinical manifestation, physical examination, enhanced computed tomography scan, histological finding, and microorganism culture of biopsied specimen in endoscopy.

**Interventions:**

The patient was treated with gastrointestinal decompression and broad-spectrum antibiotics.

**Outcomes:**

He recovered from phlegmonous gastritis and received the 2nd cycle of chemotherapy with no complaint of abdominal discomfort.

**Lessons:**

Early recognition and proper management including broad-spectrum antibiotics are key approaches to phlegmonous gastritis.

## 1 Introduction

Phlegmonous gastritis is a rare and rapidly progressive condition with a high mortality rate ranging from 26% to 64% that occurs mainly in individuals aged 30 to 70 years old.^[1–4]^ It is characterized by suppurative bacterial infection starting from the submucosal and muscular layers of the gastric wall, which is different from other forms of acute gastritis in which inflammatory changes mainly occur in the gastric mucosa. It is often seen in patients with mucosal injury, alcoholism, immunocompromised state, and malignancy, though the exact pathogenesis remains unknown.^[2,3]^ Diagnosis of phlegmonous gastritis is difficult and requires a comprehensive analysis of clinical manifestation, imaging, bacterial culture, and pathology. The typical clinical presentation of phlegmonous gastritis is the acute onset of a triad of symptoms, including epigastric pain, vomiting, and fever with or without feverish chill.^[5]^ Pus in vomitus, which is quite rare, is considered pathognomonic for the diagnosis. Echography could help with the early diagnosis of phlegmonous gastritis, revealing a thickened gastric wall.^[6]^ Computed tomography (CT) is especially useful and usually reveals a thickened gastric wall and hypodense areas within the thickened walls. An enhanced peripheral rim around the low-density areas indicative of intramural abscesses could further help the diagnosis.^[7,8]^ Gastric mucosa is usually edematous with dirty necrotic materials covering the surface under endoscopy. *Streptococcus* spp is the most common microorganism, accounting for 68% phlegmonous gastritis cases.^[2]^ Histological findings were characterized by infiltration of neutrophils and plasma cells, as well as intramural hemorrhage and necrosis. Management requires early recognition and treatment with antibiotics as soon as possible and sometimes surgery, while standard treatment remains unestablished.

Data of patients with hematological malignancies developing phlegmonous gastritis are limited. Here, we report a male patient with mixed-phenotype acute leukemia (MPAL) who developed acute phlegmonous gastritis during the neutropenia phase on induction chemotherapy and was successfully treated.

## 2 Case presentation

**Figure 1:**
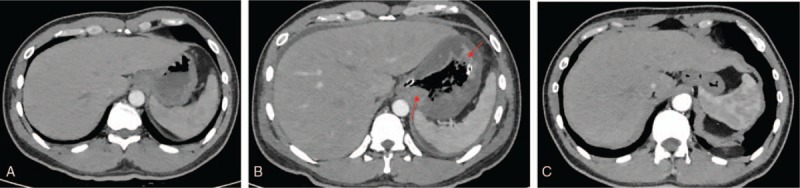
(A) Enhanced computed tomography (CT) scan on admission. No abnormalities were found. (B) Enhanced CT scan the 2nd day after phlegmonous gastritis. There is diffused mural thickening of the stomach with hypodense areas within the wall (arrows). (C) Gastric thickening improved remarkedly more than 2 months after phlegmonous gastritis.

A 33-year-old male complained of leucopenia in routine physical examination without symptoms. He had a history of chronic gastritis for 2 years. On physical examination, vital signs were stable, and enlarged superficial lymph nodes were palpated upon admission. Complete blood count (CBC) showed white blood cell (WBC) was 1.20 × 10^9^/L; hemoglobin, 122 g/L; and platelet, 134 × 10^9^/L. Bone marrow aspirate revealed hypoplasia with 62% blast cells. Immunohistochemistry showed that the blasts were positive for myeloperoxidase staining and periodic acid-Schiff staining. Flow cytometry analyses demonstrated an abnormal blast population with expression of CD34, CD7, and cCD3 and no expression of specific B lymphoid markers. Metaphase assessment showed normal karyotype. Molecular studies found positive TCRδ rearrangement. Diagnosis of MPAL, T/myeloid, not otherwise specified was made according to WHO 2016 criteria.^[9]^ No abnormality was found in enhanced chest and abdominal CT scan before induction chemotherapy (Fig. 1A). The patient received the planned induction chemotherapy, including 4 mg vindesine (iv, d1, d8, d15, and d21), 80 mg daunorubicin (45 mg/m^2^, iv, d1–d3), and 10 mg dexamethasone (equal to prednisone 1 mg/kg·m^2^, iv, d1–d21). Pantoprazole (40 mg, iv, twice daily), a proton pump inhibitor (PPI), was administered because of intermittent stomach discomfort from the beginning of chemotherapy.

**Figure 2:**
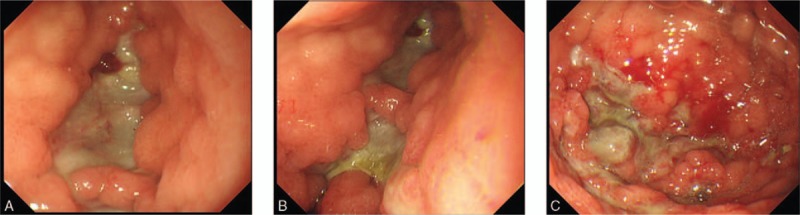
Gastroscopy revealed gastric ulcers covered with pus scattering in (A) antrum, (B) body, and (C) fundus of the stomach.

**Figure 3:**
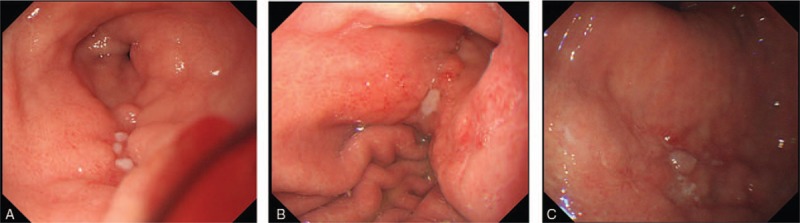
Endoscopy reperformed showed significant improvements, including much smaller ulcers and less pus in (A) antrum, (B) body, and (C) fundus of the stomach.

On day 9 during chemotherapy, the patient complained of gripping pain in the left epigastrium and heavy vomiting with gastric contents and blood clots. Vital signs were stable on examination. The abdomen was tender to palpation in the upper left quadrant, without guarding or rebound tenderness. Pantoprazole was infused continuously because of gastrointestinal hemorrhage. However, the symptoms were not relieved. CBC showed that WBC was 0.07 × 10^9^/L; hemoglobin, 96 g/L; and platelet, 32 × 10^9^/L. Prothrombin time and active partial thrombin time were normal. On day 10, he developed fever (37.9 °C) with chill, while epigastric pain got worse, and he complained of dry mouth and oliguria. On physical examination, the skin was clammy. Blood pressure was 80/50 mm Hg, heart rate was 150 to 160 beats per minute, respiratory rate was 26 breaths per minute, and oxygen saturation was 96% while he was breathing ambient air. The abdomen was tender to palpation, especially below the xiphoid. On day 10, CBC showed that WBC was 0.1 × 10^9^/L; hemoglobin, 60 g/L; and platelet, 16 × 10^9^/L. C-reactive protein was 113 mg/L (normal range <8 mg/L), and procalcitonin was 29.77 ng/mL (normal range <0.05 ng/mL). Blood culture for bacteria and fungi was negative, as reported several days later. Emergent abdominal ultrasound demonstrated diffusely thickened stomach. Gastrointestinal decompression was adopted, and bloody fluids were drained out. A diagnosis of neutropenia and shock was given, and phlegmonous gastritis was suspected. Considering dramatically decreasing hemoglobin, fever, and increasing C-reactive protein, the shock symptoms might be caused by hemorrhage and infection. Fluid resuscitation was initiated, red blood cells were transfused, and intravenous imipenem and vancomycin were empirically infused to cover possible pathogens. Blood pressure and heart rate returned to normal levels after several hours of fluid resuscitation. Abdominal pain and distension were then relieved. Chemotherapy was discontinued. On day 11, tigecycline was added, as his temperature reached as high as 39 °C. His temperature slowly decreased thereafter and went back to normal on day 14. On day 11, an enhanced CT scan was carried out and revealed diffused gastric wall thickening with hypodense areas within the wall; the diagnosis of phlegmonous gastritis was confirmed (Fig. 1B). Endoscopy was not performed because of low platelet count, ranging from 10 × 10^9^ to 25 × 10^9^/L. After a follow-up CT scan on day 17 showed a reduction of the gastric wall thickening, the gastric tube was withdrawn, and the patient was allowed liquid food. Endoscopy was performed on day 23, when his platelet count recovered to 80 × 10^9^/L, and revealed diffuse edema, as well as erythema of the gastric wall. Gastric ulcers were found scattered in the antrum, body, and fundus of the stomach, with the largest being 4 × 1.5 cm, and they were covered with pus (Fig. 2). Histological findings of the biopsied specimen showed severe inflammatory cell infiltration. Although blood culture was negative, *Stenotrophomonas maltophilia* was cultured from the biopsied specimen. The patient was placed on a normal diet on day 24 and discharged from the hospital. Endoscopy reperformed on day 51 showed there were much smaller ulcers and less pus in the stomach (Fig. 3). On day 75, the patient received the 2nd cycle of chemotherapy with reduced doses, including 640 mg cyclophosphamide (375 mg/m^2^, iv, d1 and d2), 75 mg daunorubicin (45 mg/m^2^, iv, d1−d3), 3 mg vindesine (iv, d1 and d8), and 20 mg prednisone (po, d1−d8, then reducing gradually) combined with gaster, an H2 receptor antagonist, rather than pantoprazole. He did not complain of any abdominal discomfort during the 2nd cycle of chemotherapy. An enhanced abdominal CT scan on day 93 revealed marked improvement in gastric thickening (Fig. 1C).

## 3 Discussion

Phlegmonous gastritis is a rare condition with a high mortality rate. Here, we reported a patient with MPAL who had a history of chronic gastritis and developed phlegmonous gastritis with typical symptoms and image-based and pathologic features during neutropenia phase on induction chemotherapy. *S maltophilia*, a relatively rare pathogen found in phlegmonous gastritis, was cultured from the gastroscopic biopsy specimen. He recovered by gastrointestinal decompression and broad-spectrum antibiotics.

Phlegmonous gastritis has been reported in a few patients with hematological malignancies including gastric lymphoma, myeloid sarcoma, multiple myeloma, and acute leukemia.^[10–13]^ Several risk factors, including acute leukemia, receiving chemotherapy including cytotoxic agents and corticosteroids, and being in the neutropenic phase, lead to the immunocompromised status in our patient, which contributed to the onset of phlegmonous gastritis.

Phlegmonous gastritis is caused by a bacterial infection of the gastric wall. The most commonly identified pathogens are *Streptococcus* spp, accounting for more than half of the cases,^[2,3]^ followed by *Enterococcus* spp, *Klebsiella pneumonia*, *Staphylococcus* spp, *Enterobacter cloacae*, *Clostridium* spp, and *Citrobacter freundii*. However, *S maltophilia* was cultured from the biopsied specimen of our patient, which has rarely been reported in published cases with phlegmonous gastritis and is a global emerging opportunistic environmental pathogen. We suspected that *S maltophilia* might lead to phlegmonous gastritis in our patient based on several reasons: biopsy was taken of gastric ulceration, which was covered with pus even though endoscopy was performed 2 weeks after the onset of phlegmonous gastritis; multiorgan infections caused by opportunistic pathogens are very common in patients with acute leukemia during the neutropenia phase, and *S maltophilia* cultured in the biopsied specimen from our patient is a common pathogen found in respiratory infection^[14]^; and *S maltophilia* is sensitive to tigecycline, which was used in our patient 2 days after phlegmonous gastritis was diagnosed when the body temperature continued to rise.^[15,16]^

Our patient was administered with PPI because of a history of chronic gastritis and gastric discomfort during the early phase of chemotherapy. PPI, as the name indicates, is an inhibitor of H^+^K^+^ adenosine triphosphatase, the proton pump of the parietal cell. It causes diminished H^+^ secretion into the secretory canaliculus, resulting in reduced gastric acid production and protecting gastric mucosa. Gastric acid is well known for its role as a primary bactericidal barrier. Bacterial infections have been found to be associated with a marked reduction in gastric acid secretion.^[17]^ PPI was also reported to impair neutrophil function and diminish the bactericidal effect of neutrophils.^[18]^ Patients on PPI therapy were reported to have an increased risk of bacterial enteric infections with *Clostridium difficile*, *Salmonella*, and *Campylobacter*.^[19]^ The reports about *S maltophilia* on PPI therapy were limited. Therefore, we cannot exclude the possibility that the use of PPI might have contributed to the onset of phlegmonous gastritis. We noticed that PPI use was reported in 2 cases before the onset of phlegmonous gastritis.^[20,21]^

The standard treatment of phlegmnous gastritis remained unestablished. The use of broad-spectrum of antibiotics should be initiated without delay. Surgical treatment was superior to medical treatment (mortality rate 18% vs 100%) 4 decades ago.^[4]^ However, with rapid progress in antibiotics, the outcome of medical treatment improved significantly with mortality rate decreased to 19%.^[3]^

## 4 Conclusion

We reported a case of a patient with acute leukemia who developed phlegmonous gastritis during the neutropenia phase of chemotherapy, which might have been caused by the infection of *S maltophilia*. Special attention should be paid to patients with a history of chronic gastritis and use of glucocorticoid and PPI during chemotherapy. Early recognition and proper management, including broad-spectrum antibiotics, are key approaches to this rare and fatal comorbidity.

## Author contributions

**Conceptualization:** Meng Lv, Rui Liu, Ting Zhao.

**Data curation:** Dayu Shi, Jinde He.

**Formal analysis:** Dayu Shi, Jinde He.

**Investigation:** Qian Jiang.

**Methodology:** Qian Jiang.

**Writing – original draft:** Dayu Shi, Jinde He, Qian Jiang.

**Writing – review & editing:** Qian Jiang.
